# Frequencies of PD-1 and LAG-3 positive T cells in asthmatic children and their relationship with inflammatory cytokines

**DOI:** 10.1515/med-2025-1288

**Published:** 2026-02-24

**Authors:** Jie Huang, Guoxun Zhang, Wen Yuan, Ying Tao, Haibin Yuan

**Affiliations:** Department of Paediatrics, The First People’s Hospital of Xiangtan, Xiangtan, China; Department of Laboratory Medicine, The First People’s Hospital of Xiangtan, Xiangtan, China

**Keywords:** asthma, PD-1, LAG-3, Th2 cytokines, immune checkpoint

## Abstract

**Objectives:**

This study aimed to investigate the expression of programmed cell death protein 1 (PD-1) and lymphocyte-activation gene 3 (LAG-3) on CD4^+^ and CD8^+^ T cells in children with asthma and their relationship with Th2-associated inflammatory cytokines (IL-4, IL-5, and IL-13). The goal was to elucidate the potential roles of these immune checkpoint molecules in asthma pathogenesis and severity.

**Methods:**

A prospective observational study was conducted involving 112 asthmatic children aged 5–15 years and 100 healthy controls. Peripheral blood mononuclear cells (PBMCs) were isolated, and flow cytometry was used to analyze the frequencies of PD-1^+^ and LAG-3^+^ T cells. Serum levels of IL-4, IL-5, and IL-13 were measured using ELISA. Asthma severity was classified according to the Global Initiative for Asthma (GINA) guidelines, and demographic, clinical, and lung function data were collected. Statistical analyses included Pearson correlation, ROC curve analysis, and logistic regression to assess the diagnostic and prognostic value of PD-1 and LAG-3 expression.

**Results:**

Asthmatic children, particularly those with moderate-to-severe disease, exhibited significantly higher frequencies of PD-1^+^ and LAG-3^+^ T cells compared to healthy controls. Serum levels of IL-4, IL-5, and IL-13 were also elevated in asthmatic children, with the highest levels observed in moderate-to-severe cases. The frequencies of PD-1^+^LAG-3^+^ T cells were positively correlated with IL-13 levels and negatively correlated with lung function parameters, including FVC%, FEV1%, and PEF%. ROC curve analysis demonstrated that CD4^+^PD-1^+^LAG-3^+^ T cells had superior diagnostic performance for moderate-to-severe asthma. Logistic regression identified CD4^+^LAG-3^+^PD-1^+^, and IL-13 as independent risk factors for moderate-to-severe asthma.

**Conclusions:**

The elevated frequencies of PD-1 and LAG-3 on T cells in asthmatic children, particularly in those with moderate-to-severe disease, suggested that these immune checkpoint molecules play a critical role in asthma pathogenesis and severity. These findings highlighted the potential of PD-1 and LAG-3 as biomarkers for asthma severity and therapeutic targets, offering new avenues for immune modulation in pediatric asthma management.

## Introduction

Asthma is a chronic respiratory disease that significantly impacts the quality of life, particularly in children. Epidemiological statistics indicate that asthma affects over 300 million people worldwide, and this number is projected to rise to 400 million by 2025 [1], 2]. It is characterized by recurrent episodes of wheezing, breathlessness, chest tightness, and coughing, often triggered by environmental factors such as allergens, infections, or air pollution [3], 4]. While mild asthma can often be managed with standard therapies, moderate-to-severe asthma poses a significant clinical challenge, leading to frequent exacerbations, reduced quality of life, and increased healthcare burden [5]. It has been reported that about 5–10 % of asthmatics have severe conditions [6]. Severe asthma in children is associated with persistent airway inflammation, remodeling, and irreversible lung function decline [7], highlighting the need for better biomarkers to predict disease progression and guide targeted therapies [8]. In this context, identifying immune markers associated with asthma severity and inflammation is crucial for improving disease management and outcomes.

Programmed cell death protein 1 (PD-1) and lymphocyte-activation gene 3 (LAG-3) are co-inhibitory receptors expressed on T cells that play critical roles in regulating immune responses and maintaining immune tolerance [9]. PD-1 is well-known for its role in T cell exhaustion in chronic infections and cancer, while LAG-3 is involved in suppressing T cell activation and cytokine production [10], 11]. Co-expression of PD-1 and LAG-3 on T cells has been associated with a more profound state of immune dysfunction in various diseases, including autoimmune disorders, chronic viral infections, and cancer [12], [13], [14]. However, their roles in allergic diseases, particularly asthma, remain poorly understood. Emerging evidence suggested that alterations in the PD-1 pathway could modulate the Treg/Th17 balance in children with asthma [15], while LAG-3 regulated antigen-specific immune responses to suppress airway inflammation [16].

Given the limited understanding of PD-1 and LAG-3 in pediatric asthma, this study aims to investigate the expression of PD-1 and LAG-3 on CD4^+^ and CD8^+^ T cells in asthmatic children and their relationship with Th2-associated inflammatory cytokines (IL-4, IL-5, and IL-13). By comparing healthy controls, mild asthmatics, and moderate-to-severe asthmatics, we seek to elucidate the potential roles of these markers in asthma pathogenesis and severity.

## Methods

### Study population

This prospective observational study included 112 children with asthma who were admitted to the Department of Pediatrics at our hospital from May 2023 to February 2025. The diagnosis of asthma was established based on the Global Initiative for Asthma (GINA) guidelines [17], which include a history of recurrent wheezing, cough, and reversible airflow limitation confirmed by spirometry with a positive bronchodilator response (an increase in forced expiratory volume in 1 s [FEV1] of ≥12 % and 200 mL post-bronchodilator administration). All children participating in the study are aged between 5 and 15 years. Those with cystic fibrosis, bronchiectasis, or other chronic respiratory diseases, as well as children suffering from severe infections, hematological disorders, malignant tumors, or compromised heart, liver, or kidney function, are excluded from the study. Additionally, children with immunodeficiencies, autoimmune diseases, or those who have recently undergone immunosuppressive therapy are also excluded. Furthermore, the classification of asthma severity in children was conducted according to the “Guidelines for the Diagnosis and Management of Bronchial Asthma in Children (2016 edition) [18]” which divided them into categories of mild asthma and moderate to severe asthma. Additionally, the identification of all pediatric asthma cases as allergic asthma was informed by methodologies established in prior research [19].

100 Age- and sex-matched healthy controls were recruited from the general pediatric population during routine health check-ups. Controls were required to have no history of asthma, allergic diseases, or other chronic illnesses, and no recent infections or use of medications that could influence immune function.

### Flow cytometry

Peripheral blood samples (approximately 5 mL) were collected from all study participants, including both asthmatic children and healthy controls, via venipuncture of the antecubital vein using EDTA-coated tubes to prevent coagulation. Peripheral blood mononuclear cells (PBMCs) were isolated using density gradient centrifugation with Ficoll-Paque PLUS (GE Healthcare, USA) according to the manufacturer’s instructions [20], 21]. Briefly, whole blood was diluted with phosphate-buffered saline (PBS) at a 1:1 ratio, layered over Ficoll-Paque, and centrifuged at 400×g for 30 min at room temperature. The PBMC layer was carefully collected, washed twice with PBS, and resuspended in complete RPMI-1640 medium supplemented with 10 % fetal bovine serum (FBS) and 1 % penicillin-streptomycin.

For flow cytometry analysis, PBMCs were stained with fluorochrome-conjugated antibodies against surface markers to identify T cell subsets. The following antibodies were used: anti-CD4 (FITC), anti-CD8 (APC), anti-PD-1 (PE), and anti-LAG-3 (BV421) (Abcam, USA). Cells were incubated with the antibodies for 30 min at 4 °C in the dark, followed by two washes with PBS. Flow cytometry was performed using a BD FACSCanto II flow cytometer (BD Biosciences, USA), and data were analyzed using FlowJo software (version 10.8.1, TreeStar). The frequencies of PD-1^+^ and LAG-3^+^ cells within CD4^+^ and CD8^+^ T cell populations were determined and compared between asthmatic children and healthy controls.

### Measurement of serum inflammatory markers by ELISA

Serum levels of inflammatory cytokines, including interleukin-4 (IL-4), interleukin-5 (IL-5), and interleukin-13 (IL-13), were measured using commercially available enzyme-linked immunosorbent assay (ELISA) kits according to the manufacturer’s instructions. Briefly, venous blood samples were collected from all study participants (asthmatic children and healthy controls) in serum separator tubes and allowed to clot at room temperature for 30 min. The samples were then centrifuged at 1,500×g for 10 min to separate the serum, which was aliquoted and stored at −80 °C until analysis.

For the ELISA assays, serum samples were thawed on ice, and all reagents were brought to room temperature prior to use. Standards and samples were added to the pre-coated wells in duplicate, followed by the addition of biotin-conjugated detection antibodies specific to IL-4, IL-5, and IL-13. After incubation and washing to remove unbound substances, streptavidin-horseradish peroxidase (HRP) was added to each well. A substrate solution (tetramethylbenzidine, TMB) was then added to induce a colorimetric reaction, which was stopped by adding a sulfuric acid stop solution. The optical density (OD) of each well was measured at 450 nm using a microplate reader (BioTek Instruments, USA). The concentrations of IL-4, IL-5, and IL-13 in the serum samples were calculated based on the standard curves generated for each cytokine. The kit manufacturers as well as the sensitivity and detection ranges of the assays were as follows: IL-4 (MBS2020632, USA, sensitivity: 5.9 pg/mL, range: 15.6–1,000 pg/mL), IL-5 (MBS2023393, USA, sensitivity: 6.4 pg/mL, range: 15.6–1,000 pg/mL), and IL-13 (MBS2019436, USA, sensitivity: 6.7 pg/mL, ange: 5–500 pg/mL). All procedures were performed in accordance with the manufacturer’s protocols, and appropriate quality controls were included in each assay run to ensure accuracy and reproducibility.

### Collection of demographic and clinical parameters

Demographic and clinical data were systematically collected for all study participants, including both asthmatic children and healthy controls. Demographic information included age, sex, body mass index (BMI), history of allergies, family history of asthma or other allergic diseases, and environmental exposure factors (passive smoking and pet ownership). Clinical parameters specific to asthma patients were also recorded, including disease duration, frequency of asthma exacerbations in the past year.

Lung function tests were performed using a spirometer (CareFusion MasterScreen, Germany) according to the American Thoracic Society (ATS) and European Respiratory Society (ERS) guidelines. The following parameters were measured: forced vital capacity (FVC) % predictive, forced expiratory volume in 1 s (FEV1) % predictive, peak expiratory flow (PEF) % predictive, and the FEV1/FVC % ratio. Each participant was instructed to perform at least three acceptable maneuvers, and the highest value was recorded for analysis.

### Statistical analysis

Data analysis was performed using SPSS 26.0 statistical software. The Kolmogorov-Smirnov test was employed to confirm the normal distribution of the data. Normally distributed data are presented as mean ± standard deviation (SD), while non-normally distributed data are expressed as median (range). Comparisons between two groups were conducted using the Mann-Whitney *U* test or the Student’s t-test, as appropriate. One-way analysis of variance (ANOVA) followed by Tukey’s post hoc test was used for comparison among three groups. Proportions were analyzed using the chi-square test. Pearson correlation analysis was utilized to assess the relationships between circulating T cell subtypes, inflammatory cytokines, and pulmonary function parameters. To evaluate the diagnostic value of circulating T cell subtypes for children with asthma and those with moderate to severe asthma, ROC curve analysis was performed. Additionally, multivariable logistic regression analysis was conducted to identify risk factors for moderate to severe asthma. A p-value of <0.05 was considered statistically significant.

### Ethical approval

Written informed consent was obtained from the parents or legal guardians of all participants, and the study protocol was approved by the Institutional Review Board of our hospital (approval number: 2024111902).

## Results

### Basic characteristics of the study participants

The demographic and clinical characteristics of the study participants are summarized in Tables 1 and 2. In Table 1, asthmatic children were compared with healthy controls regarding demographic data. Asthmatic children showed a significantly higher prevalence of allergic history and family history of asthma compared to healthy controls. No significant differences were observed in age, sex distribution, BMI, or environmental exposure factors such as passive smoking and pet ownership between the two groups. In Table 2, asthmatic children were stratified into mild (n=43) and moderate-to-severe groups (n=69) based on the GINA classification. Moderate-to-severe asthmatic children exhibited significantly lower lung function parameters, including FVC% predicted, FEV1% predicted, and FEV1/FVC ratio, compared to mild asthmatics (p<0.05). Additionally, moderate-to-severe asthmatic children had a higher frequency of asthma exacerbations in the past year compared to mild asthmatics.

**Table 1:** Basic characteristics between asthmatic children and healthy children.

Variable	Asthmatic children, n=112	Healthy children, n=100	p-Value
Age, y	10 (5–15)	9 (5–15)	0.261
Sex, female, %	52 (46.4)	50 (50.0)	0.610
BMI	18.94 (13.93–22.25)	18.39 (13.89–22.17)	0.429
Family history of asthma, n (%)	42 (37.5)	2 (2.0)	<0.001
Family history of allergies, n (%)	51 (45.5)	17 (17.0)	<0.001
Smoking environment, n (%)	29 (25.9)	24 (24.0)	0.756
Pet environment, n (%)	21 (18.8)	18 (18.0)	0.884

**Table 2:** Basic characteristics and clinical data between mid and moderate-to-severe asthmatic children.

Variable	Mid asthmatic children, n=43	Moderate-to-severe asthmatic children, n=69	p-Value
Age, y	10 (5–15)	10 (5–15)	0.646
Sex, female, %	19 (44.2)	33 (47.8)	0.610
BMI	18.64 (13.93–22.00)	19.12 (14.23–22.25)	0.776
Family history of asthma, n (%)	15 (34.9)	27 (39.1)	0.538
Family history of allergies, n (%)	19 (44.2)	32 (46.4)	0.755
Smoking environment, n (%)	13 (30.2)	16 (23.2)	0.263
Pet environment, n (%)	7 (16.3)	14 (20.3)	0.464
Disease duration, y	2 (1–3)	4 (1–7)	<0.001
Asthma exacerbations past year	1 (0–2)	5 (2–8)	<0.001
FVC % predictive	76.68 ± 4.07	66.60 ± 4.73	<0.001
FEV1 % predictive	79.80 ± 7.44	63.77 ± 9.69	<0.001
PEF % predictive	74.88 ± 4.31	63.97 ± 4.84	<0.001
FEV1/FVC, % predictive	69.88 ± 5.85	60.88 ± 5.61	<0.001

### Expression of inflammatory cytokines and PD-1/LAG-3-positive T cell frequencies in asthmatic children

**Figure 1:**
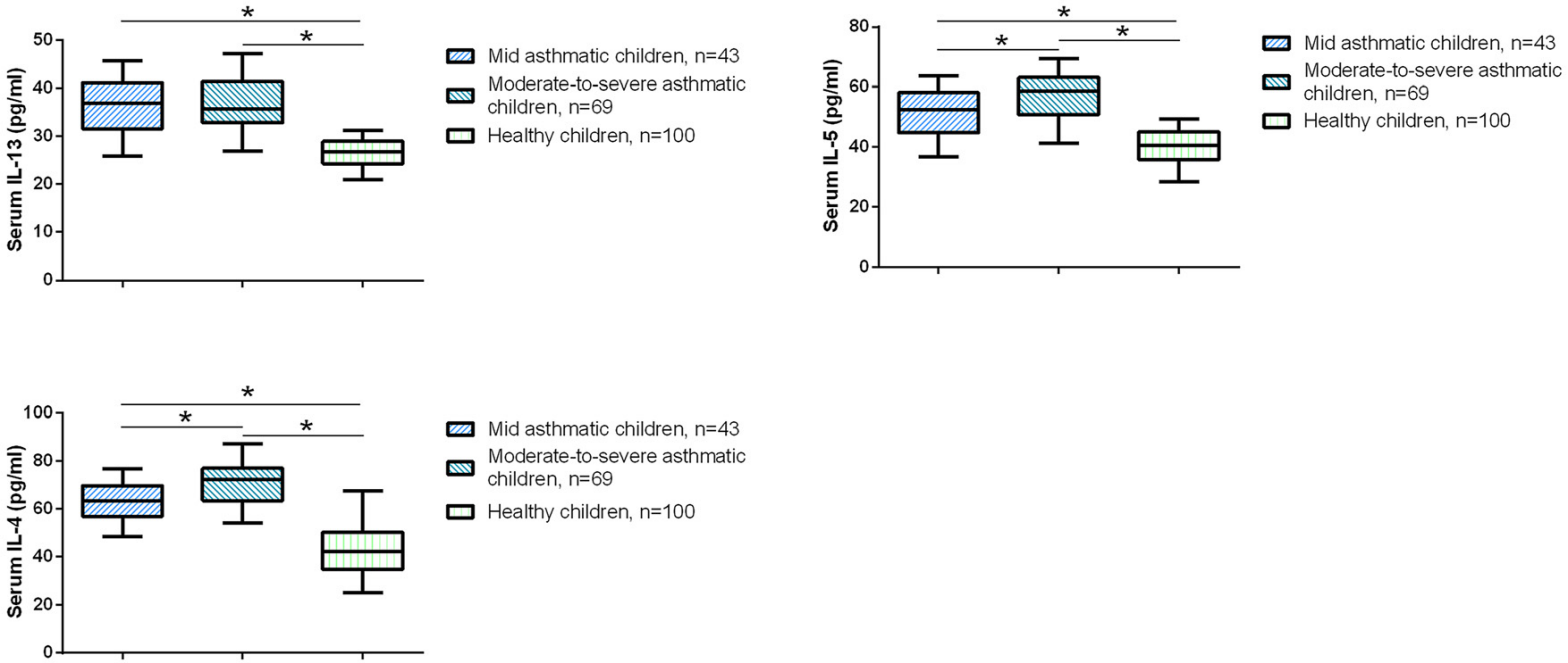
Serum levels of Th2-associated cytokines in asthmatic children and healthy controls. Serum IL-13, IL-5, and IL-4 levels were measured in mild asthmatic children (n=43), moderate-to-severe asthmatic children (n=69), and healthy children (n=100) using ELISA. *p<0.05.

The levels of inflammatory cytokines (IL-4, IL-5, and IL-13) and the frequencies of PD-1 and LAG-3-positive T cell subsets were compared among the three study groups: healthy controls, mild asthmatic children, and moderate-to-severe asthmatic children. The serum levels of IL-4, IL-5, and IL-13 were significantly elevated in asthmatic children compared to healthy controls, with the highest levels observed in moderate-to-severe asthmatics. This pattern is consistent with previous studies reporting elevated Th2 cytokines in asthma and their association with disease severity [22], 23]. Specifically, IL-4 levels were significantly elevated in asthmatic children compared to healthy controls; however, no significant difference was observed between mild and moderate-to-severe asthma groups (Figure 1, p<0.05). In contrast, IL-5 and IL-13 levels exhibited a stepwise increase across the three groups, with the lowest levels observed in healthy children, intermediate levels in those with mild asthma, and the highest levels in moderate-to-severe asthmatics; these differences were all statistically significant (p<0.05). These findings suggest a Th2-skewed inflammatory response that intensifies with asthma severity.

**Figure 2:**
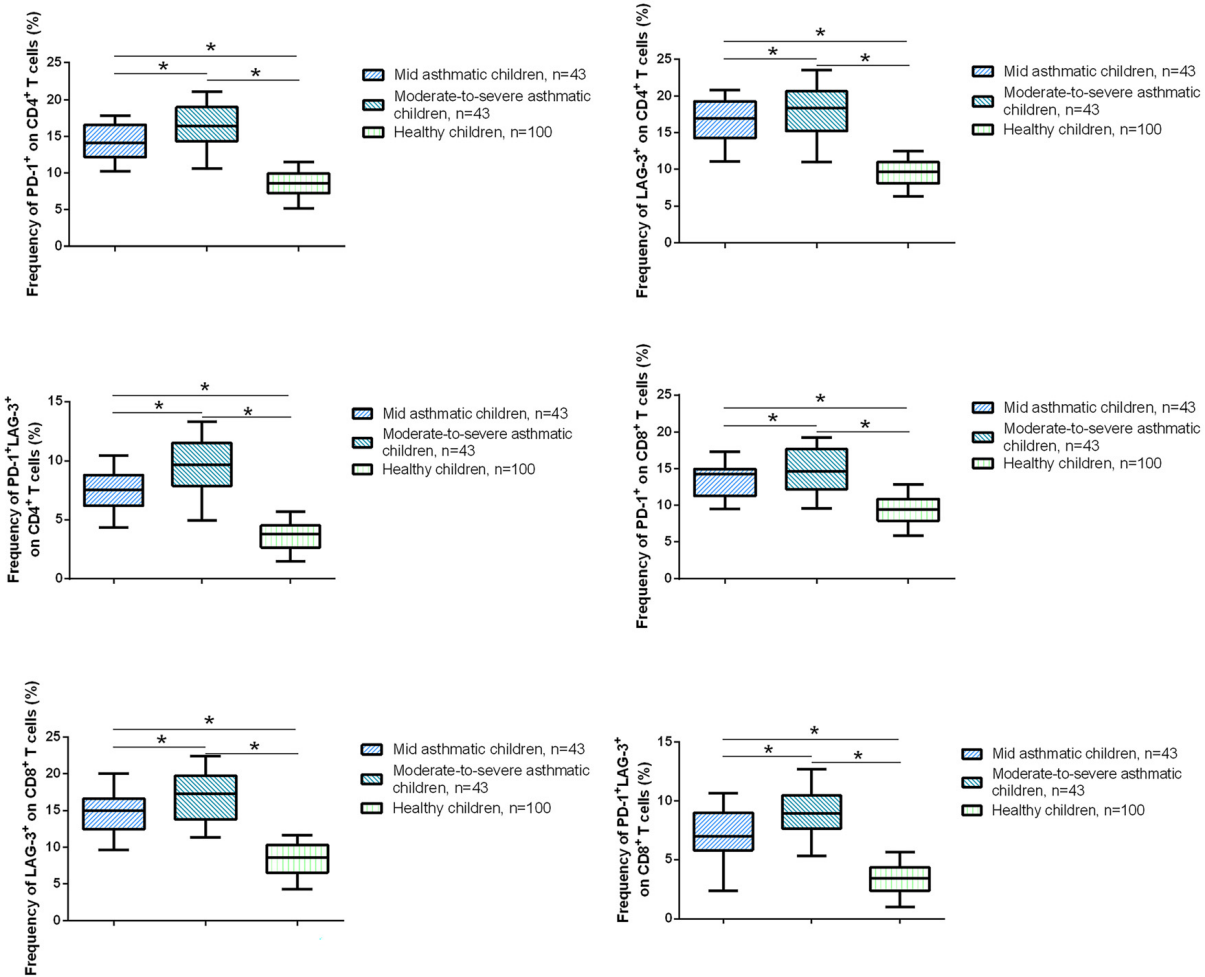
Frequencies of PD-1^+^ and LAG-3^+^ T cell subsets in peripheral blood from asthmatic children and healthy controls. Flow cytometry was used to analyze the frequencies of PD-1^+^ and LAG-3^+^ cells within CD4^+^ and CD8^+^ T cell populations in mild asthmatic children (n=43), moderate-to-severe asthmatic children (n=69), and healthy children (n=100). *p<0.05.

In CD4^+^ T cells, the frequencies of PD-1^+^ and LAG-3^+^ subsets were significantly higher in asthmatic children compared to healthy controls, with the highest frequencies observed in moderate-to-severe asthmatics. Notably, the frequency of CD4^+^PD-1^+^LAG-3^+^ double-positive T cells was also elevated in asthmatic children, particularly in the moderate-to-severe group. Similarly, in CD8^+^ T cells, the frequencies of PD-1^+^, LAG-3^+^, and PD-1^+^LAG-3^+^ subsets were significantly increased in asthmatic children, with the most pronounced differences seen in moderate-to-severe asthmatics (Figure 2, p<0.05). Representative flow cytometry data showing the gating strategy for identifying CD4^+^ or CD8^+^ T cells and the distribution of PD-1/LAG-3 single- and double-positive populations in moderate-to-severe asthmatic children are presented for CD4^+^PD-1^+^LAG-3^+^ (Supplementary Figure 1) and CD8^+^PD-1^+^LAG-3^+^ (Supplementary Figure 2). These results indicate that both CD4^+^ and CD8^+^ T cells exhibit enhanced expression of inhibitory receptors (PD-1 and LAG-3) in asthma, which correlates with disease severity.

### Association of PD-1^+^, LAG-3^+^, and PD-1^+^LAG-3^+^ T cell subtypes with cytokines and pulmonary function

**Figure 3:**
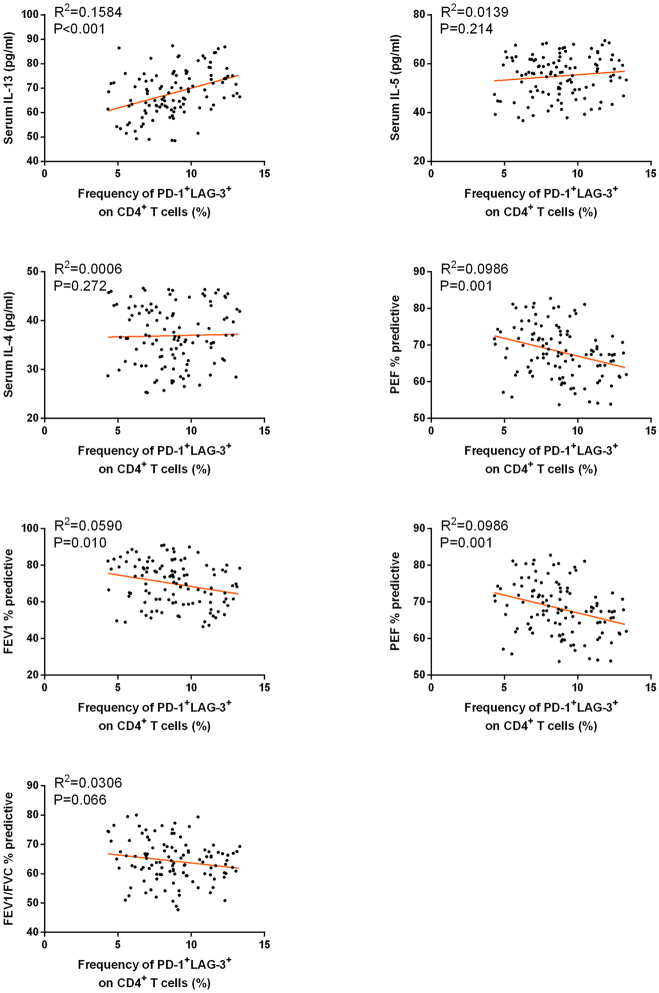
Correlations between the frequency of CD4^+^PD-1^+^LAG-3^+^ T cells and serum cytokine levels (IL-13, IL-5, IL-4) and pulmonary function parameters (PEF% predicted, FEV_1_% predicted, FEV_1_/FVC% predicted) in moderate-to-severe asthmatic children.

**Figure 4:**
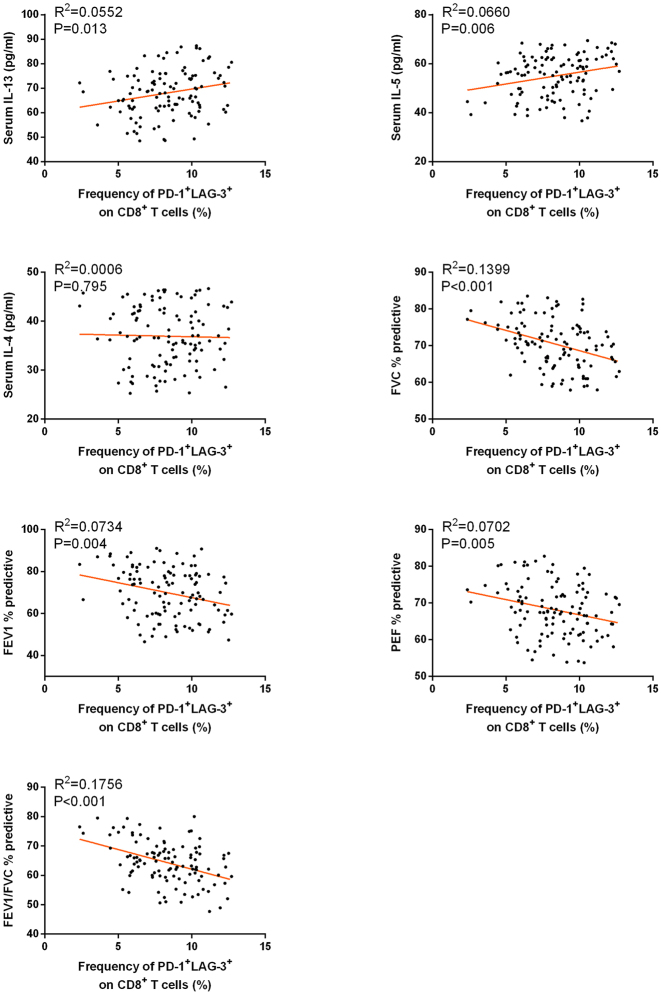
Correlations between the frequency of CD8^+^PD-1^+^LAG-3^+^ T cells and serum cytokine levels (IL-13, IL-5, IL-4) and pulmonary function parameters (FVC% predicted, FEV_1_% predicted, PEF% predicted, FEV_1_/FVC% predicted) in moderate-to-severe asthmatic children.

We further investigated the relationship between positive T cell subtypes in children with asthma and levels of inflammatory cytokines, as well as pulmonary function parameters, through Pearson correlation analysis. As shown in Table 3, the analysis revealed that the frequencies of CD4^+^PD-1^+^LAG-3^+^ and CD8^+^PD-1^+^LAG-3^+^ cells were positively correlated with IL-13 levels. Additionally, the frequency of CD4^+^PD-1^+^LAG-3^+^ and CD8^+^PD-1^+^LAG-3^+^ cells was negatively correlated with the pulmonary function parameters FVC % predicted, FEV1 % predictive and PEF % predicted. The correlations between the frequency of CD4^+^PD-1^+^LAG-3^+^ or CD8^+^PD-1^+^LAG-3^+^ T cells with serum cytokine levels and pulmonary function parameters were shown as scatter plots in Figures 3 and 4. These findings indicate that the frequency of PD-1^+^LAG-3^+^ positive T cells is associated with inflammatory responses and pulmonary function in children with asthma.

**Table 3:** Correlation analysis among circulating T cell frequencies and inflammatory factors and pulmonary function in asthma children.

	CD4^+^			CD8^+^		
	PD-1^+^	LAG-3^+^	PD-1^+^LAG-3^+^	PD-1^+^	LAG-3^+^	PD-1^+^LAG-3^+^
IL-13						

Pearson’s correlation	0.319	0.137	0.398	0.180	0.110	0.235
p	0.001	0.149	<0.001	0.058	0.247	0.013

IL-5						

Pearson’s correlation	0.111	0.051	0.118	0.143	0.361	0.257
p	0.245	0.593	0.214	0.132	<0.001	0.006

IL-4						

Pearson’s correlation	−0.007	0.160	0.025	0.105	0.019	−0.025
p	0.942	0.092	0.795	0.272	0.846	0.795

FVC % predictive						

Pearson’s correlation	−0.223	−0.151	−0.334	−0.145	−0.252	−0.374
p	0.018	0.113	<0.001	0.128	0.007	<0.001

FEV1 % predictive						

Pearson’s correlation	−0.228	−0.109	−0.243	−0.279	−0.181	−0.271
p	0.016	0.252	0.010	0.003	0.056	0.004

PEF % predictive						

Pearson’s correlation	−0.306	−0.097	−0.314	−0.078	−0.166	−0.265
p	0.001	0.308	0.001	0.413	0.080	0.005

FEV1/FVC, % predictive						

Pearson’s correlation	−0.379	−0.310	−0.175	−0.159	−0.154	−0.419
p	<0.001	0.001	0.066	0.094	0.105	<0.001

**Figure 5:**
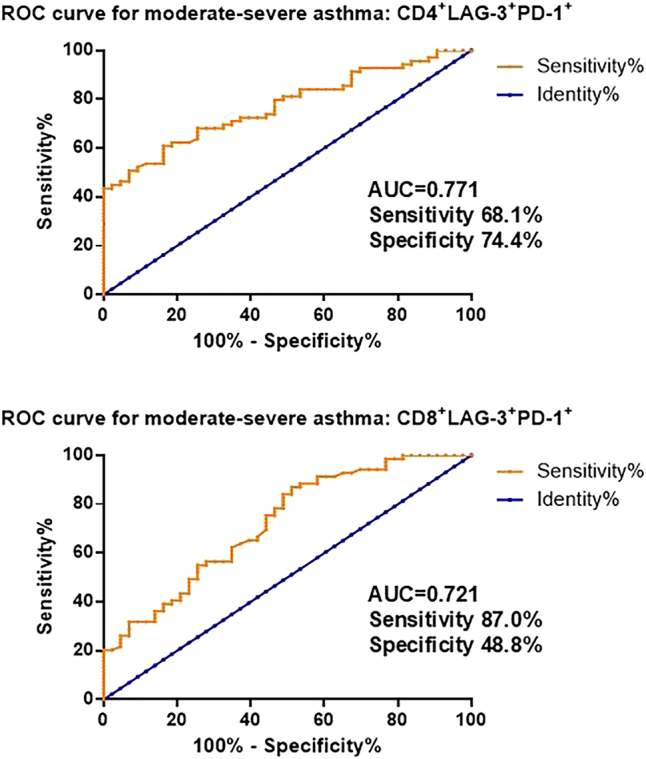
Receiver operating characteristic (ROC) curve analysis of circulating PD-1^+^LAG-3^+^ T cells for the diagnosis of moderate-to-severe asthma.

### Diagnostic value of circulating T cell subtypes in children with moderate to severe asthma

Receiver operating characteristic (ROC) curve analysis was conducted to further evaluate the diagnostic value of PD-1^+^LAG-3^+^ T cell subtypes in identifying moderate-to-severe asthma in children. As shown in Figure 5, both CD4^+^PD-1^+^LAG-3^+^ and CD8^+^PD-1^+^LAG-3^+^ T cell subsets demonstrated diagnostic potential. The CD8^+^PD-1^+^LAG-3^+^ T cell subset yielded an AUC of 0.721, with a cutoff value of 6.65 %, sensitivity of 87.0 %, and specificity of 48.8 %. Notably, the CD4^+^PD-1^+^LAG-3^+^ T cell subset exhibited superior diagnostic performance, with an AUC of 0.771, a cutoff value of 8.73 %, sensitivity of 68.1 %, and specificity of 74.4 %.

### Risk factor analysis

We conducted a binary logistic regression analysis to assess the risk factors for children with moderate to severe asthma. The analysis results indicated that CD4^+^LAG-3^+^PD-1^+^, and IL-13 were identified as independent risk factors for children with moderate to severe asthma (Table 4).

**Table 4:** Logistic regression of risk factors for moderate-severe asthma.

Variables	Wald	Odds ratio	95 % CI	p-Value
Age, y	0.055	1.022	0.854–1.222	0.814
Sex	0.269	1.396	0.395–4.929	0.604
BMI	0.415	1.110	0.807–1.527	0.520
Family history of asthma	0.536	1.614	0.448–5.822	0.464
Family history of allergies	0.515	1.574	0.456–5.434	0.473
Smoking environment	2.971	0.302	0.077–1.179	0.085
Pet environment	0.001	0.990	0.290–3.381	0.988
IL-13	3.935	1.081	1.001–1.169	0.047
IL-5	2.205	1.069	0.979–1.168	0.138
IL-4	0.117	1.017	0.923–1.121	0.732
CD4^+^PD-1^+^	2.885	1.242	0.966–1.598	0.091
CD4^+^LAG-3^+^	1.651	1.131	0.937–1.366	0.199
CD4^+^PD-1^+^ LAG-3^+^	7.445	1.706	1.162–2.505	0.006
CD8^+^PD-1^+^	1.903	1.198	0.927–1.550	0.168
CD8^+^LAG-3^+^	3.785	1.238	0.998–1.534	0.052
CD8^+^PD-1^+^ LAG-3^+^	2.403	1.286	0.936–1.767	0.121

## Discussion

Asthma, particularly in pediatric populations, where it contributes to substantial morbidity, reduced quality of life, and increased healthcare costs. Moderate-to-severe asthma, in particular, is associated with persistent inflammation, airway remodeling, and irreversible lung function decline, underscoring the need for better biomarkers to predict disease progression and guide therapeutic interventions [24]. This study investigated the expression of PD-1 and LAG-3 on T cells and their relationship with Th2-associated inflammatory cytokines (IL-4, IL-5, and IL-13) in asthmatic children. Our findings reveal that both PD-1 and LAG-3-positive T cells are significantly elevated in asthmatic children, particularly in those with moderate-to-severe disease, suggesting their potential role in asthma pathogenesis and severity. These results highlight the importance of immune checkpoint molecules in asthma and provide a foundation for further exploration of their therapeutic potential.

In recent years, numerous studies have begun to explore the role of serum biomarkers in asthma, highlighting their potential in understanding disease mechanisms and guiding clinical management. Baioumy et al. demonstrated that serum zonulin, a marker of intestinal permeability, is significantly elevated in asthmatic patients and correlates with asthma severity, suggesting its potential as a prognostic factor [25]. Similarly, Abdullah et al. found that serum myeloperoxidase (MPO) levels are significantly higher in asthmatic patients compared to healthy controls, although its correlation with asthma control remains unclear [26]. Yavuz et al. reported that serum periostin levels are elevated in children with asthma, particularly in those with severe disease, although its diagnostic utility for severe asthma is limited [27]. Moreover, Wang et al. concluded that children with asthma have lower serum 25-hydroxyvitamin D (25-OHD) levels compared to healthy children, and vitamin D supplementation can reduce asthma recurrence rates [28]. These findings collectively underscore the importance of serum biomarkers in asthma research and their potential clinical applications. In this study, we observed significantly elevated levels of Th2-associated cytokines (IL-4, IL-5, and IL-13) in asthmatic children compared to healthy controls, with the highest levels found in moderate-to-severe asthmatics. These findings are consistent with previous studies demonstrating that Th2 inflammation plays a central role in asthma pathogenesis, particularly in allergic asthma. IL-4 and IL-13 are known to promote IgE production and airway hyperresponsiveness, while IL-5 is critical for eosinophil recruitment and activation [29], 30]. The progressive increase in these cytokines from mild to moderate-to-severe asthma suggests that Th2 inflammation intensifies with disease severity, aligning with other reports that have linked higher cytokine levels to worse clinical outcomes. Our results support the use of these cytokines as biomarkers for monitoring asthma severity and guiding targeted therapies, such as biologics that specifically inhibit Th2 pathways.

The pathogenesis of asthma is closely linked to dysregulated immune responses, particularly involving T cell-mediated inflammation. In allergic asthma, the immune system overreacts to environmental triggers, leading to the activation of Th2 cells and the release of pro-inflammatory cytokines [31]. These cytokines drive eosinophilic inflammation, airway hyperresponsiveness, and mucus production, which are hallmark features of asthma [32]. Additionally, regulatory T cells (Tregs) and their associated immune checkpoint molecules play a critical role in maintaining immune homeostasis and preventing excessive inflammation. However, in chronic inflammatory conditions like asthma, the balance between immune activation and suppression is often disrupted, resulting in persistent airway inflammation and tissue remodeling [33]. Immune checkpoint molecules, such as PD-1 and LAG-3, are key regulators of T cell function, and their dysregulation has been implicated in various immune-mediated diseases, including asthma. In our research, the elevated frequencies of PD-1 and LAG-3-positive T cells in asthmatic children, particularly in moderate-to-severe cases, suggest that these inhibitory receptors may play a role in modulating immune responses in asthma.

PD-1 and LAG-3 are well-known for their roles in T cell exhaustion and immune tolerance. In chronic hepatitis B, elevated PD-1 and LAG-3 expression on CD4+ T cells correlates with disease progression and impaired cytokine production, which can be partially restored by blocking these pathways [14]. In chronic kidney disease, increased frequencies of PD-1^+^ and LAG-3^+^ T cells are associated with elevated inflammatory cytokines and poor prognosis, highlighting their potential as therapeutic targets [34]. In rheumatoid arthritis, soluble LAG-3 levels are elevated in both early and chronic stages, correlating with autoantibody seropositivity and radiographic progression, while LAG-3 functionally suppresses inflammatory cytokine production [35]. In asthma, our findings suggest that these receptors may similarly modulate T cell responses, potentially leading to chronic inflammation and impaired immune regulation. The co-expression of PD-1 and LAG-3 on both CD4^+^ and CD8^+^ T cells further underscores their synergistic role in immune dysfunction.

The up-regulation of PD-1 and LAG-3 expression in asthma may be attributed to chronic exposure to environmental allergens and sustained airway inflammation, leading to persistent activation of T cells and dysregulated immune responses [36]. Recent studies have demonstrated that the PD-1/PD-L1 axis plays a context-dependent regulatory role in allergic asthma. In a murine model of allergic asthma, blockade of PD-1 aggravated airway hyperresponsiveness by shifting the immune response toward a Th17 phenotype, and PD-1 signaling modulates T cell responses in allergic inflammation in a subset-specific manner [37]. PD-1 and its ligands (PD-L1 and PD-L2) are involved in regulating IgE-mediated responses and Th2 cell activation in allergic asthma, and these immune checkpoint pathways may function as inhibitory brakes or permissive modulators of adaptive immunity [38]. Moreover, Helou et al. demonstrated that PD-1 functions as a metabolic checkpoint in group 2 innate lymphoid cells (ILC2s), limiting their viability and effector functions, and that PD-1 agonist treatment alleviates airway hyperreactivity and lung inflammation in allergic asthma [39]. Similarly, LAG-3 has been implicated in modulating antigen-specific immune tolerance. It was shown to suppress airway inflammation through regulatory T cell mechanisms and via double-negative T cells in mouse models [16]. Taken together, these findings highlight the complex and context-specific roles of PD-1 and LAG-3 in regulating allergic airway inflammation. Rather than functioning solely as markers of T cell exhaustion, these molecules may actively contribute to shaping the immune milieu in asthma by modulating effector cell function, maintaining immune tolerance, and limiting excessive inflammation. This mechanistic framework provides biological plausibility for the involvement of PD-1 and LAG-3 in asthma pathogenesis. Their co-expression on T cells may represent a compensatory response to chronic allergen-induced immune activation.

In addition to PD-1 and LAG-3, other immune checkpoint molecules have also been implicated in asthma and allergic diseases. CTLA-4 regulated early T cell activation by competitively binding to B7 ligands, thereby suppressing T cell costimulation and downstream Th2 cytokine production. Blockade of the CD28–B7 pathway by CTLA4-IgG in a murine asthma model effectively attenuated eosinophilic infiltration, airway hyperresponsiveness, and IL-4 secretion [40]. TIM-3 expression was upregulated in CD4^+^ T cells following allergen challenge and likely modulated asthma-related inflammation by repressing Th1 responses and shifting the immune balance toward Th2 dominance [41]. TIGIT was involved in asthma through T cell exhaustion mechanisms driven by transcription factors such as c-Maf and Blimp-1 [42]. Similar to these immune checkpoint molecules, PD-1 and LAG-3 are also involved in controlling T cell exhaustion and chronic inflammation [43], 44]. These findings underscore the relevance of PD-1 and LAG-3 in asthma pathogenesis, where chronic allergen exposure and ongoing immune activation contribute to airway inflammation and disease progression. Our observation of elevated PD-1 and LAG-3 expression in T cells from asthmatic children, especially in severe cases, underscores their potential role as biomarkers of disease severity and as candidates for future immunomodulatory interventions.

While this study provides valuable insights into the role of PD-1, LAG-3, and Th2 cytokines in pediatric asthma, several limitations should be acknowledged. First, the observed associations do not establish a definitive causal relationship between these immune markers and asthma severity. Future longitudinal studies are warranted to clarify whether increased PD-1 and LAG-3 expression contributes to disease progression or results from chronic airway inflammation. Second, the relatively small sample size limits the generalizability of our findings and may not fully reflect the heterogeneity of asthma phenotypes. Validation in larger, multicenter cohorts is essential to confirm the observed associations and enhance the robustness of our conclusions. Finally, while we focused on Th2 cytokines, other inflammatory pathways, such as Th17 and innate immunity, may also contribute to asthma pathogenesis and warrant further investigation.

## Conclusions

Our research demonstrated that PD-1 and LAG-3-positive T cells were significantly elevated in asthmatic children, particularly in those with moderate-to-severe disease, and are associated with increased levels of Th2 cytokines. These findings suggested that immune checkpoint molecules might play a critical role in asthma pathogenesis and severity, offering potential targeted for therapeutic intervention. Future studies should explore the mechanisms underlying PD-1 and LAG-3 regulation in asthma and evaluate the efficacy of immune checkpoint modulation in improving clinical outcomes. By identifying novel biomarkers and therapeutic targets, this research contributes to the growing body of knowledge aimed at improving the management of pediatric asthma.

## Supplementary Material

Supplementary Material

Supplementary Material

Supplementary Material
