# Supplementary figures and images for: Frequencies of PD-1 and LAG-3 positive T cells in asthmatic children and their relationship with inflammatory cytokines
Source: Open Med (Wars). 2026 Feb 24;21(1):20251288. doi: 10.1515/med-2025-1288 (PMC12927456; doi:10.1515/med-2025-1288)

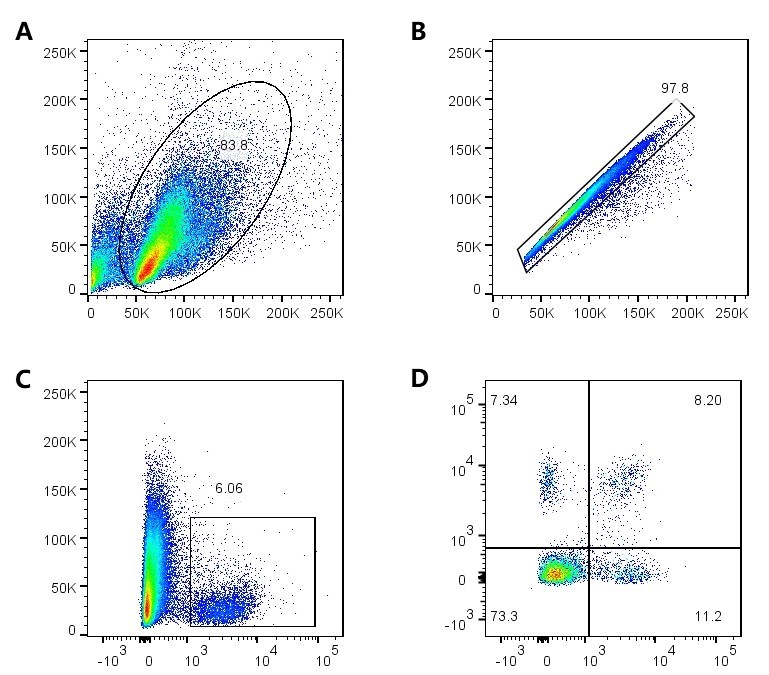

Supplement: Supplementary file 1 — Supplementary Material [file j_med-2025-1288_suppl_001.jpg]

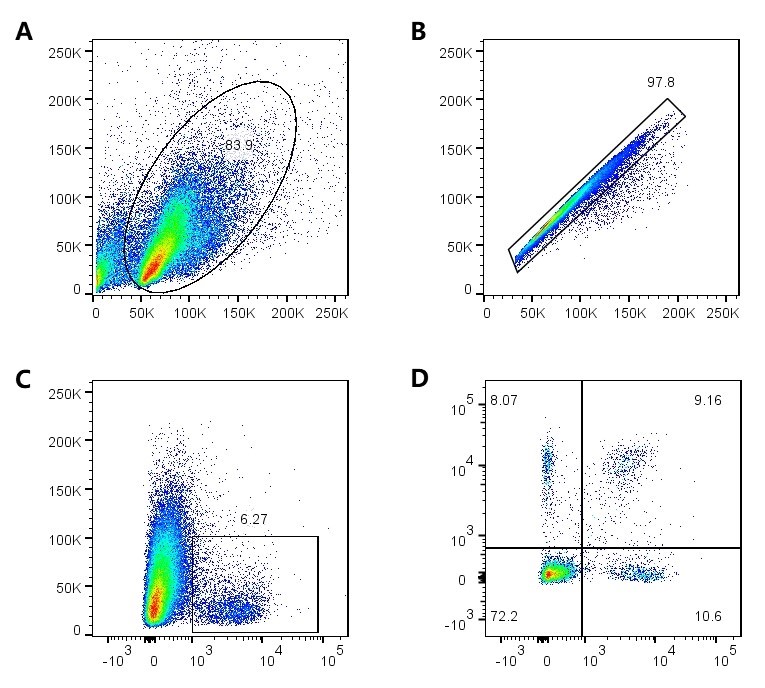

Supplement: Supplementary file 2 — Supplementary Material [file j_med-2025-1288_suppl_002.jpg]
