# Supplementary material for: Frequencies of PD-1 and LAG-3 positive T cells in asthmatic children and their relationship with inflammatory cytokines
Source: Open Med (Wars). 2026 Feb 24;21(1):20251288. doi: 10.1515/med-2025-1288 (PMC12927456; doi:10.1515/med-2025-1288)
Supplement: Supplementary file 3 — Supplementary Material [file j_med-2025-1288_suppl_003.docx]

Supplementary Figure 1. **Representative flow cytometry plots of CD4^+^PD-1^+^LAG-3^+^ cells in moderate-to-severe asthmatic children.** (A) Forward scatter (FSC) versus side scatter (SSC) plot to select lymphocytes. (B) FSC-A versus FSC-H plot to exclude doublets. (C) CD4 expression to identify CD4^+^ T cells. (D) PD-1 versus LAG-3 expression profile to determine PD-1 single-positive, LAG-3 single-positive, and PD-1/LAG-3 double-positive CD4^+^ T cell populations.

Supplementary Figure 2. **Representative flow cytometry plots of CD8^+^PD-1^+^LAG-3^+^ cells in moderate-to-severe asthmatic children.** (A) Forward scatter (FSC) versus side scatter (SSC) plot to select lymphocytes. (B) FSC-A versus FSC-H plot to exclude doublets. (C) CD8 expression to identify CD8^+^ T cells. (D) PD-1 versus LAG-3 expression profile to determine PD-1 single-positive, LAG-3 single-positive, and PD-1/LAG-3 double-positive CD8^+^ T cell populations.
